# Accuracy in Quantitative Electron Probe Microanalysis

**DOI:** 10.6028/jres.093.139

**Published:** 1988-06-01

**Authors:** K. F. J. Heinrich

**Affiliations:** National Bureau of Standards, Gaithersburg, MD 20899

Electron probe microanalysis (EPMA) is based on the interpretation of x-ray spectra emitted by specimens which were exposed to a focused, accelerated electron beam. One of the attractions of this technique is the simplicity of the x-ray emission line spectra which is particularly striking if we do not pay attention to the fine details of x-ray spectrometry such as line position and shape changes with chemical composition or the extended structure of absorption edges. In close analogy, the inventor of EPMA, R. Castaing, found in early investigations that quite simple approximations to quantitation with this technique could yield results of remarkable analytical accuracy, considering the state of art of instrumentation at that time, and the small volumes from which the compositional analysis was elicited [1]. While Castaing favored a simple approach based on physical principles, Ziebold and Ogilvie [2] chose an empirical technique based on the use of composite standard materials; again, a pleasing simplicity was observed, and the empirical method is still widely used, particularly in the analysis of minerals.

As experience and areas of applications widened, it became obvious that to increase the accuracy of the procedure, some of this apparent simplicity had to be abandoned. Fortunately, the availability of small computers permitted the on-line execution of more involved data reduction schemes; larger computers could be used in Monte Carlo simulations of the events in the target [3], and the quality of estimates of pertinent parameters, such as the x-ray absorption coefficients, was also improved [4].

The x-ray line spectra observed in EPMA are caused by electrons which penetrate the specimen surface with energies which typically range between 5 and 30 keV. In the typical flat, thick specimen, most of the electron paths are contained within a homogeneous specimen matrix. The penetrating electrons lose energy due to inelastic interactions which usually are treated as a continuous process and described by Bethe’s law of electron deceleration or by expressions related to this law [4]. The direction of the penetrating electron is altered mainly due to inelastic collisions; the scattering of electrons causes a significant number of electrons to be re-emitted from the specimen (backscattering), and the fraction of electrons which are backscattered depends strongly on the mean atomic number of the target.

The backscattered electrons conserve most of the energy they had when entering the specimen; therefore, a significant fraction of the energy which would otherwise cause x-ray emission is lost through backscattering, and the loss varies strongly with specimen composition.

A small fraction of electron-target interactions, described by ionization cross-sections produces x-rays, and the x-ray emission directed toward the specimen surface suffers attenuation which is determined by the distribution in depth of the loci of excitation and by the x-ray absorption coefficients of the specimen.

Besides this primary x-ray generation, processes such as fluorescent x-ray generation by continuous as well as characteristic x-rays affect the relation between x-ray emission intensity and specimen composition. It is common, though not justifiable by present standards of computation, to group the data processing steps into three multiplicative factors (Z, A, F), which, respectively, represent the “atomic number correction” (i.e., consideration of electron deceleration and backscatter), the “absorption correction” (which only considers the attenuation of primary x-rays), and the “fluorescence correction” (which describes, usually in an oversimplified way, the generation and attenuation of x-rays excited by other characteristic x-ray lines). The fluorescence due to the continuum is ignored in most data reduction schemes.

In addition to the uncertainties in parameters involved in the aforementioned processes, the accuracy of the procedure is affected by lack of flatness and homogeneity of the standards whose emission is compared with that of the specimen, by errors in the determination of the composition of nonelemental standards, and by statistical and systematic errors in the measurement of x-ray lines and in the separation of line intensity from spectral background. The errors in estimating the characteristics of the detector system, with exception of deadtime effects, cancel when the emission from the specimen is divided by that from the standard. The estimate of achievable accuracy of EPMA and the comparison of diverse approaches must therefore involve consideration of theoretical aspects, of errors in the values of parameters used in the procedure, and of operational errors in specimen and standard preparation and in analysis. In the development of current “correction procedures,” theoretical, mathematical and measurements aspects are interwoven. For instance, the distribution in depth of x-ray generation can either be calculated by Monte Carlo calculations, which are limited by the accuracy of available parameters, or by exeriments with sandwich tracer targets. To further complicate things, some Monte Carlo calculations use techniques empirically adjusted to fit the available experimental evidence. Since the precision of measurement of relative x-ray intensities is better than the accuracy of results on specimens of known composition, there is room for improvement; but, to achieve this, the diverse potential source of errors will have to be unraveled and tested separately.
